# Early Radiological Response Following Concurrent Chemoradiotherapy and Brachytherapy in Cervical Carcinoma: A Single-Centre Observational Study From Pakistan

**DOI:** 10.7759/cureus.116908

**Published:** 2026-09-25

**Authors:** Anam Siddique, Nusrat Nazar, Mohammad A Sahi, Jannat Asif, Taleeha Moazam, Abdul S Waqar

**Affiliations:** 1 Oncology, Cancer Care Hospital & Research Centre, Lahore, PAK

**Keywords:** brachytherapy, cervical cancer, concurrent chemoradiotherapy, figo staging, radiological response, recist 1.1

## Abstract

Background: Cervical cancer remains an important cause of cancer morbidity and mortality, with a disproportionate burden in low- and middle-income countries. Definitive treatment for non-metastatic disease requiring radical therapy generally combines external-beam radiotherapy, concurrent platinum-based chemotherapy, and brachytherapy. This study primarily aimed to determine the distribution of complete response, partial response, stable disease, and progressive disease on routine pelvic magnetic resonance imaging performed approximately three months after treatment and secondarily to explore whether objective response differed by recorded FIGO (International Federation of Gynecology and Obstetrics) 2009 stage group.

Methods: This retrospective single-centre observational study was conducted in the Department of Clinical Oncology, Cancer Care Hospital & Research Centre, Lahore, Pakistan, and included 113 women with histologically confirmed cervical carcinoma, recorded as FIGO 2009 stage IB1 to IVA, who completed planned external-beam radiotherapy and brachytherapy and had follow-up pelvic MRI available at approximately three months. The institutional protocol comprised external-beam radiotherapy to 45 Gy in 25 fractions using conventional two-dimensional simulation, with weekly cisplatin planned at 40 mg/m², followed by CT-planned high-dose-rate intracavitary brachytherapy at 7 Gy per fraction for three fractions. Investigators retrospectively mapped routine radiology-report descriptions to complete response, partial response, stable disease, or progressive disease using RECIST 1.1 concepts; this was not a formal prospectively measured or blinded central RECIST assessment. The primary outcome was the four-category early radiological response distribution. Objective response was defined as complete plus partial response. The stage comparison was exploratory and unadjusted.

Results: The mean age was 50.8 ± 10.2 years. Squamous cell carcinoma was the predominant histology (82/113, 72.6%). Complete response occurred in 61 patients (54.0%; 95% CI 44.8-62.9%), partial response in 32 (28.3%), stable disease in 14 (12.4%), and progressive disease in six (5.3%). Objective response occurred in 93 patients (82.3%; 95% CI 74.2-88.2%). In the exploratory stage analysis, objective response occurred in 77 of 78 patients (98.7%) in the FIGO IB1-IIB group and 16 of 35 patients (45.7%) in the FIGO IIIB-IVA group (absolute difference 53.0 percentage points; 95% CI 36.0-68.3; Fisher's exact test, p < 0.001).

Conclusion: This study provides real-world insight into early MRI-assessed radiological response following definitive chemoradiotherapy and brachytherapy for cervical cancer in a resource-constrained Pakistani setting. The observed difference in response between FIGO stage groups was exploratory and requires confirmation in future studies. Longer follow-up is needed to determine whether early radiological response translates into durable disease control and survival.

## Introduction

Cervical cancer remains a major global health problem and disproportionately affects women in low- and middle-income countries (LMICs) [1]. Globally, an estimated 604,000 new cervical cancer cases and approximately 280,000 deaths occurred in 2024 [1]. In Pakistan, the 2023 Catalan Institute of Oncology (ICO)/International Agency for Research on Cancer (IARC) human papillomavirus (HPV) Information Centre fact sheet estimated approximately 5,008 new cervical cancer cases and 3,197 deaths annually; cervical cancer was reported as the third most frequent cancer among women and the second most frequent among women aged 15-44 years [2]. These figures underscore the relevance of reporting treatment outcomes from Pakistani oncology services, particularly where organized screening and access to contemporary radiotherapy infrastructure may be limited.

For patients with non-metastatic cervical carcinoma requiring definitive treatment, cisplatin-based concurrent chemoradiotherapy followed by brachytherapy remains a standard radical treatment approach [3,4]. Contemporary European Society of Gynaecological Oncology (ESGO)/European Society for Radiotherapy and Oncology (ESTRO)/European Society of Pathology (ESP) guidance favors image-based external-beam planning, preferably intensity-modulated radiation therapy (IMRT) and volumetric-modulated arc therapy (VMAT), and image-guided adaptive brachytherapy, preferably MRI-based, with target-volume and organ-at-risk dose reporting [4]. However, real-world practice in resource-constrained settings may use less advanced techniques. Outcomes from such cohorts remain clinically relevant, but they should be interpreted within their treatment context rather than as direct comparisons with modern image-guided series.

Early post-treatment imaging can provide useful information on initial tumour response, although it cannot substitute for durable locoregional control, progression-free survival, or overall survival. Pakistani series have reported short-term response after both conventional two-dimensional chemoradiotherapy and image-guided hybrid brachytherapy, but standardized early radiological-response data remain limited [5,6]. We therefore sought to determine the proportions of patients categorized as complete response, partial response, stable disease, and progressive disease on routine pelvic MRI approximately three months after treatment. A secondary exploratory objective was to compare objective response, defined as complete plus partial response, between recorded FIGO (International Federation of Gynecology and Obstetrics) 2009 stage groups. Because this was an exploratory retrospective analysis, no confirmatory directional hypothesis was specified.

## Materials and methods

Study design, setting, and ethics

This retrospective single-centre observational study was conducted in the Department of Clinical Oncology, Cancer Care Hospital & Research Centre, Lahore, Pakistan. The Institutional Review Board approved the study (IRB/CCH&RC/0102/24). The study period extended from 26 February 2024 to 31 December 2024. Patients treated during this period were screened against the predefined eligibility criteria, and follow-up pelvic MRI reports were reviewed after treatment completion. No formal sample-size or power calculation was performed because the study used all eligible patients available within the defined study period.

Study population

Analytical inclusion criteria were as follows: newly diagnosed histologically confirmed cervical carcinoma, a source-recorded FIGO 2009 stage from IB1 to IVA, completion of the planned external-beam radiotherapy and brachytherapy course, and an available follow-up pelvic MRI report at approximately three months after treatment. No specific histological subtype was prespecified for inclusion; eligible patients were required to have histologically confirmed cervical carcinoma. The histological subtypes observed in the analytical cohort are reported in the Results section. Patients were excluded from the analytical cohort if they had distant metastatic disease, had received previous oncological treatment for cervical cancer, did not complete the planned radiotherapy/brachytherapy course, or did not have the required follow-up MRI available. Suitability for radical treatment was determined by the treating oncology team after clinical assessment and baseline investigations. The cohort should therefore be regarded as a treatment-completer/follow-up-imaging cohort rather than an intention-to-treat population.

A total of 327 patients were recorded as having been assessed for eligibility, and 113 entered the final analytical cohort. The retained study dataset did not permit a reliable reason-specific breakdown for the 214 patients who were not included; this limits assessment of selection mechanisms and has been acknowledged as a limitation. The participant flow is shown in Figure 1.

**Figure 1:**
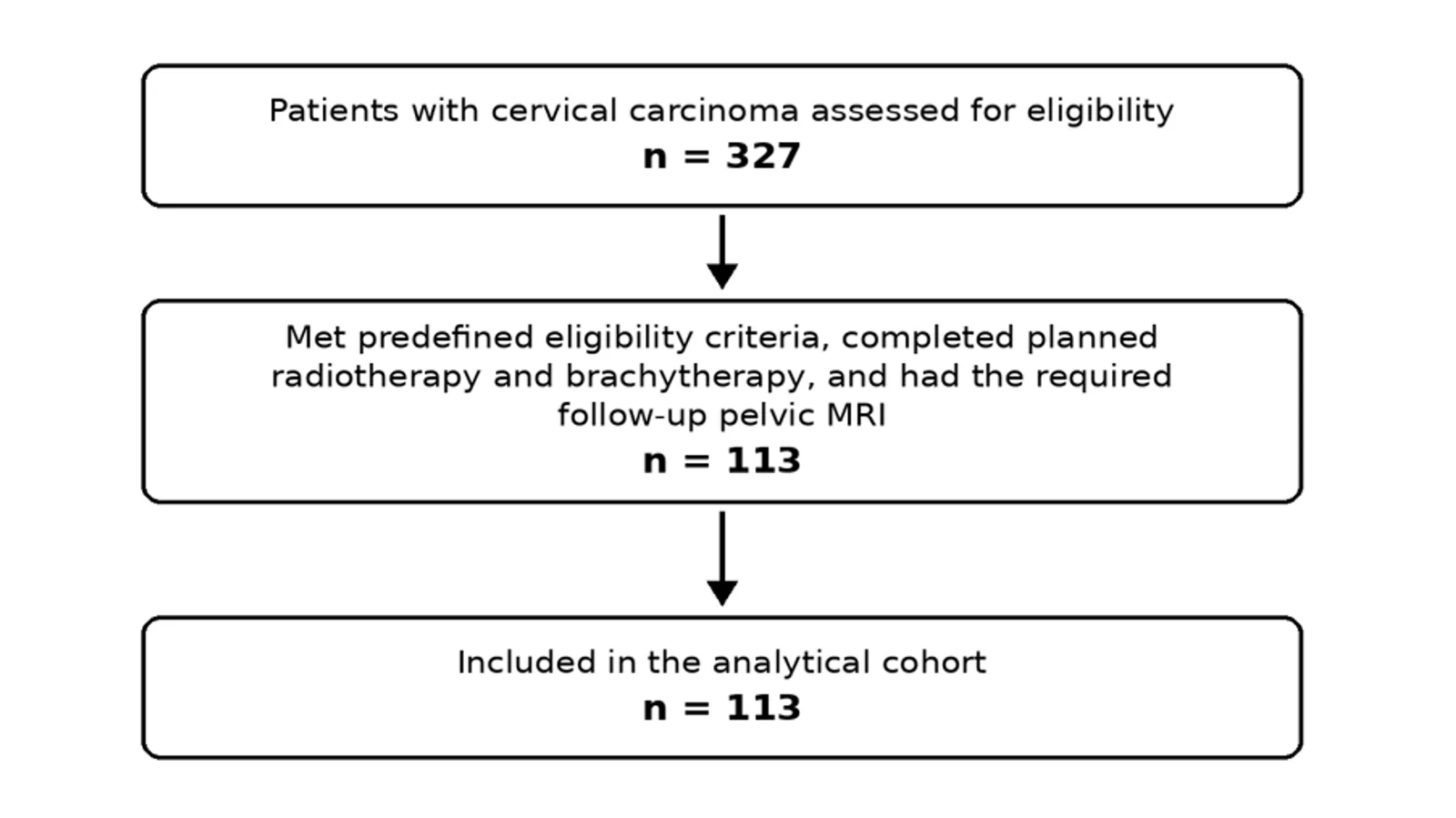
Participant flow through the study. Of 327 patients assessed for eligibility, 113 met the analytical eligibility criteria and were included in the final cohort. A reason-specific breakdown for the 214 patients not included was not available in the retained study information.

Pretreatment assessment and staging

Pretreatment assessment included clinical examination, contrast-enhanced pelvic MRI, computed tomography of the chest and abdomen, and baseline laboratory assessment for cisplatin fitness. Additional investigations, including bone scintigraphy, were performed when clinically indicated. The source records documented cervical cancer stage using FIGO 2009, and the recorded FIGO 2009 stage was therefore retained for analysis [7]. Although FIGO staging was revised in 2018, retrospective conversion was not attempted because the analytical dataset did not contain sufficiently complete tumour-size and nodal-status information to assign the revised stage reliably, particularly the FIGO 2018 stage IB size subdivisions and stage IIIC nodal categories [8]. Re-staging with incomplete data could therefore have introduced misclassification. No stage IIIA cases were recorded. For stage-wise presentation, all records within stage IIA were presented as a single IIA category because the available analytical dataset did not retain a consistently usable subdivision for all IIA cases.

The analytical dataset did not contain sufficiently complete patient-level data for several clinically relevant baseline descriptors and prognostic variables, including presenting complaints, pretreatment tumour dimensions/cT subcategories, nodal status, haemoglobin concentration, HIV status, and ECOG/WHO performance status. These variables therefore could not be summarized, stratified, or included as covariates in adjusted analyses.

Treatment

The institutional definitive treatment protocol comprised external-beam radiotherapy (EBRT) to 45 Gy in 25 fractions using conventional two-dimensional simulation, with concurrent cisplatin planned at 40 mg/m² weekly during EBRT. All 113 patients completed the planned EBRT and high-dose-rate intracavitary brachytherapy components. The exact number of cisplatin cycles actually administered to each individual patient was not available in the analytical dataset and was not analysed. Consequently, the manuscript describes the chemotherapy component as planned treatment and does not assume that every patient received the same cumulative cisplatin exposure.

The retained analytical information did not include sufficient technical detail to reconstruct external-beam field arrangement, target-volume definitions, machine/beam characteristics, patient-level boost indications or doses, or radiotherapy quality-assurance metrics. Additional external-beam boost treatment was used in selected patients when clinically indicated, but patient-level details were not available for analysis. These omissions limit reproducibility and direct comparison with contemporary image-guided techniques.

Following EBRT, high-dose-rate intracavitary brachytherapy was delivered at 7 Gy per fraction for three fractions using CT simulation/planning. The available study information did not include the applicator type, formal adaptive target definitions (for example, residual gross tumour volume, high-risk clinical target volume, or intermediate-risk clinical target volume), cumulative EQD2 target doses, or organ-at-risk dose-volume parameters such as bladder, rectum, sigmoid, or bowel D2cc. Therefore, the treatment cannot be verified from this dataset as meeting formal GEC-ESTRO/EMBRACE image-guided adaptive brachytherapy reporting criteria, and CT planning alone should not be interpreted as proof of adaptive brachytherapy [4].

Response assessment and outcomes

Follow-up pelvic MRI was performed as part of routine clinical care at approximately three months after treatment completion. The exact interval from treatment completion to MRI was not retained as an analyzable continuous variable; therefore, a median, range, or prespecified ±day window cannot be reported.

Investigators retrospectively reviewed the corresponding institutional radiology reports and mapped the report descriptions to complete response, partial response, stable disease, or progressive disease using RECIST version 1.1 concepts [9]. This process should be considered a radiology-report-derived RECIST-informed classification rather than a formal RECIST 1.1 assessment: target lesions and serial measurements were not prospectively prespecified, and no dedicated blinded central radiological re-review was performed. The investigators did not independently re-interpret the MRI images to distinguish post-radiation inflammation, oedema, or fibrosis from viable residual tumour; classification relied on the interpretation documented by the reporting radiologist in routine clinical care. Accordingly, post-treatment change may have contributed to response misclassification.

The primary outcome was the four-category early radiological response distribution. Objective response, defined as complete plus partial response, was a derived secondary outcome. The comparison of objective response between the non-overlapping FIGO IB1-IIB and FIGO IIIB-IVA groups was exploratory and unadjusted. Toxicity was not an outcome of the study because systematically graded acute and late toxicity data were not available for analysis.

Statistical analysis

Data were analysed using IBM SPSS Statistics version 25 (IBM Corp., Armonk, NY). Continuous variables were summarized as mean ± standard deviation, and categorical variables as frequencies and percentages. Wilson 95% confidence intervals were calculated for the complete-response and objective-response proportions from the reported aggregate counts. Detailed response by recorded FIGO stage was presented descriptively. Fisher's exact test was used for the exploratory 2 × 2 comparison of objective response between FIGO IB1-IIB and FIGO IIIB-IVA because of the very small number of non-responders in the FIGO IB1-IIB group. The absolute difference in response proportions and its 95% confidence interval were calculated using the Newcombe method based on Wilson score intervals to emphasize effect size rather than the p-value alone. A two-sided p-value < 0.05 was considered statistically significant.

No multivariable model was fitted because sufficiently complete patient-level covariates were not available for reliable adjustment. Histology-specific response could not be validly reconstructed from the available aggregate information and was therefore not analysed inferentially. The stage comparison should consequently be interpreted as a descriptive, hypothesis-generating association.

## Results

Baseline characteristics

A total of 113 patients comprised the analytical cohort. The mean age was 50.8 ± 10.2 years. Squamous cell carcinoma was the predominant histology, occurring in 82 patients (72.6%), followed by adenocarcinoma in 23 (20.4%) and adenosquamous carcinoma in eight (7.1%). Stage IIA was the largest recorded FIGO stage group (41/113, 36.3%). Baseline characteristics are summarized in Table 1.

**Table 1:** Baseline characteristics of the analytical cohort FIGO, International Federation of Gynecology and Obstetrics; SD, standard deviation

Variable	Category	n / summary	%
Age, years	Mean ± SD	50.8 ± 10.2	-
Histology	Squamous cell carcinoma	82	72.6
Histology	Adenocarcinoma	23	20.4
Histology	Adenosquamous carcinoma	8	7.1
FIGO 2009 stage	IB1	10	8.8
FIGO 2009 stage	IB2	11	9.7
FIGO 2009 stage	IIA	41	36.3
FIGO 2009 stage	IIB	16	14.2
FIGO 2009 stage	IIIB	14	12.4
FIGO 2009 stage	IVA	21	18.6

Early radiological response

At approximately three months after treatment completion, complete response was observed in 61 patients (54.0%; 95% CI 44.8-62.9%), partial response in 32 (28.3%), stable disease in 14 (12.4%), and progressive disease in six (5.3%). Objective response, defined as complete plus partial response, occurred in 93 of 113 patients (82.3%; 95% CI 74.2-88.2%). The four mutually exclusive response categories are summarized in Table 2.

**Table 2:** Early radiological response approximately three months after treatment Objective response was defined as complete plus partial response: 93/113 (82.3%; 95% CI 74.2-88.2%).

Response category	n	%
Complete response	61	54.0
Partial response	32	28.3
Stable disease	14	12.4
Progressive disease	6	5.3

Exploratory response by FIGO stage

Objective response was observed in all patients recorded as stage IB1, IB2, or IIB and in 40 of 41 patients recorded as stage IIA. The objective-response proportion was lower in the stage IIIB and IVA groups. Detailed stage-wise objective response is shown in Table 3. In the exploratory comparison of the non-overlapping recorded ranges FIGO IB1-IIB and FIGO IIIB-IVA, objective response occurred in 77 of 78 patients (98.7%) and 16 of 35 patients (45.7%). The absolute difference was 53.0 percentage points (95% CI 36.0-68.3), and Fisher's exact test showed p < 0.001. This estimate is unadjusted and should not be interpreted as an independent effect of stage.

**Table 3:** Objective response by the recorded FIGO 2009 stage Objective response = complete response + partial response. No objective response = stable disease + progressive disease. No stage IIIA cases were recorded. The exploratory unadjusted FIGO IB1-IIB versus FIGO IIIB-IVA comparison used Fisher's exact test (p < 0.001).

FIGO 2009 stage	Objective response, n (%)	No objective response, n (%)	Total, n
IB1	10 (100.0)	0 (0.0)	10
IB2	11 (100.0)	0 (0.0)	11
IIA	40 (97.6)	1 (2.4)	41
IIB	16 (100.0)	0 (0.0)	16
IIIB	5 (35.7)	9 (64.3)	14
IVA	11 (52.4)	10 (47.6)	21
Total	93 (82.3)	20 (17.7)	113

## Discussion

This retrospective single-centre study describes early radiological response in 113 patients with cervical carcinoma who completed planned radiotherapy and brachytherapy and had follow-up MRI available. Complete response was observed in 54.0%, partial response in 28.3%, and objective response in 82.3%. The distinction between complete and partial response is clinically important, as the broader objective-response rate should not be considered equivalent to complete tumour disappearance.

A strength of this study is that it provides real-world data on early MRI-assessed radiological response following definitive chemoradiotherapy and brachytherapy for cervical cancer in a resource-constrained Pakistani setting. By reporting the full spectrum of early response together with stage-wise descriptive outcomes in 113 treatment-completing patients, the study provides an institutional benchmark that may support future prospective research. In particular, longer-term studies are needed to determine whether early radiological response is associated with durable locoregional control, progression-free survival, and overall survival.

The observed response pattern is broadly consistent with, but not directly comparable to, other chemoradiotherapy series. A recent Pakistani cohort treated with conventional two-dimensional external-beam radiotherapy reported complete response in 42.7% and partial response in 36.2% of patients [5], while a smaller Pakistani prospective series using image-guided hybrid brachytherapy reported complete response in 65.9% and partial response in 29.3% [6]. Peng et al. reported a short-term objective-response rate of 85% in a contemporary locally advanced cervical cancer cohort [10]. Our 54.0% complete-response rate falls between the two Pakistani reports, but these differences cannot be attributed to radiotherapy technique alone because the cohorts differ in stage distribution, eligibility, brachytherapy approach, chemotherapy completion, response methodology, and follow-up timing.

These findings are particularly relevant in the global oncology context. Cervical cancer burden is concentrated in LMICs, where shortages in radiotherapy infrastructure, trained personnel, and brachytherapy capacity can constrain delivery of contemporary techniques [1,11]. Current ESGO/ESTRO/ESP guidance favors IMRT/VMAT for external-beam treatment and image-guided adaptive brachytherapy with target and organ-at-risk dose reporting [4]. The use of conventional two-dimensional external-beam simulation in this cohort reflects the real-world treatment environment in which these patients were managed and should be considered when comparing these outcomes with contemporary image-guided series. Similarly, although brachytherapy was CT-planned, available treatment data were insufficient to establish whether contemporary adaptive brachytherapy planning aims were met. These findings should therefore be interpreted as real-world outcomes from this institutional setting rather than as evidence that older and modern treatment techniques are equivalent.

Histology may also influence outcomes. In this cohort, 72.6% of patients had squamous cell carcinoma, 20.4% adenocarcinoma, and 7.1% adenosquamous carcinoma. A systematic review and meta-analysis has reported poorer disease-free and overall survival for adenocarcinoma/adenosquamous carcinoma compared with squamous cell carcinoma after definitive radiotherapy or chemoradiotherapy [12]. However, the present study does not provide a valid histology-specific response analysis and therefore cannot determine whether early radiological response differed by subtype. Future studies with patient-level linkage between histology, baseline prognostic variables, treatment delivery, and outcomes would help address this question.

The exploratory stage analysis showed a large unadjusted difference in objective response between FIGO IB1-IIB and FIGO IIIB-IVA. This association may reflect greater disease extent in later stages, but it cannot be separated from potential confounding by tumour size, nodal status, haemoglobin, performance status, treatment duration, chemotherapy exposure, histology, and other factors that were not available for adjustment. The stage comparison should therefore be interpreted as hypothesis-generating rather than as evidence that stage independently caused poorer early response.

Treatment-delivery factors may also have influenced early response. Although weekly cisplatin at 40 mg/m² was planned, individual cumulative chemotherapy exposure and overall treatment time could not be evaluated. These factors should be incorporated into future prospective studies assessing determinants of early and long-term response.

MRI provided a practical modality for early post-treatment response assessment in this cohort. However, post-radiation inflammation, oedema, and fibrosis can complicate the distinction between treatment-related change and residual tumour [13]. Because response categories were derived from routine institutional radiology reports rather than a standardized blinded central re-review, future prospective studies should incorporate predefined imaging criteria and standardized radiological assessment.

These results should not be used to justify treatment intensification, de-escalation, or deviation from current cervical cancer guidelines. Rather, their principal value is in providing real-world early response data from a Pakistani oncology centre and generating questions for future prospective research. Further studies should examine whether early MRI response is associated with longer-term disease control and survival and should incorporate more complete treatment-delivery, histological, clinical, and imaging data.

Limitations

This study has several important limitations. It was retrospective and single-centre, and the analytical cohort included only patients who completed radiotherapy and brachytherapy and underwent follow-up MRI, creating treatment-completer and complete-case selection bias. Important baseline and prognostic variables, including presenting complaints, tumour size, nodal status, haemoglobin, HIV status, and performance status, were unavailable or incomplete. For the same reason, reliable retrospective conversion from source-recorded FIGO 2009 staging to FIGO 2018 was not possible.

Treatment-delivery data were also incomplete, including individual cisplatin cycle counts, overall treatment time, detailed boost information, and radiotherapy quality-assurance parameters. The use of conventional two-dimensional EBRT limits direct comparison with contemporary IMRT/VMAT series. Brachytherapy was documented as CT-planned intracavitary HDR at 7 Gy × 3, but applicator type, adaptive target definitions, cumulative EQD2, and organ-at-risk dose-volume parameters were unavailable. Acute and late toxicity were not systematically recorded.

Response assessment was retrospective, based on routine radiology reports without blinded central review or prospectively standardized RECIST measurements, and the exact MRI interval was not retained. Histology-specific response and multivariable analysis could not be performed. Finally, the short follow-up does not permit assessment of locoregional control, progression-free survival, overall survival, or late toxicity.

## Conclusions

This study provides real-world insight into early MRI-assessed radiological response following definitive chemoradiotherapy and brachytherapy for cervical cancer in a resource-constrained Pakistani setting. The exploratory stage-wise findings provide a basis for further investigation but should not be interpreted as independent prognostic effects. Prospective studies with standardized imaging assessment and longer follow-up are needed to determine whether early radiological response translates into durable locoregional control, progression-free survival, and overall survival.
